# Synthesis of Photoswitchable Magnetic Au–Fullerosome Hybrid Nanomaterials for Permittivity Enhancement Applications

**DOI:** 10.3390/molecules200814746

**Published:** 2015-08-13

**Authors:** Min Wang, Seaho Jeon, Chefu Su, Tzuyang Yu, Loon-Seng Tan, Long Y. Chiang

**Affiliations:** 1 Department of Chemistry, Institute of Nanoscience and Engineering Technology, University of Massachusetts Lowell, Lowell, MA 01854, USA; E-Mails: wangmin81@gmail.com (M.W.); seaho_jeon@uml.edu (S.J.); 2 Department of Civil and Environmental Engineering, University of Massachusetts Lowell, Lowell, MA 01854, USA; E-Mails: CheFu_Su@student.uml.edu (C.S.); TzuYang_Yu@uml.edu (T.Y.); 3 Functional Materials Division, AFRL/RXA, Air Force Research Laboratory, Wright-Patterson Air Force Base, Dayton, OH 45433, USA; E-Mail: Loon-Seng.Tan@wpafb.af.mil

**Keywords:** fullerenyl chromophore conjugates, gold-fullerosome hybrid nanomaterials, core-shell nanoparticles, permittivity, relative dielectric constant enhancement

## Abstract

We designed and synthesized several nanomaterials **3** of three-layered core-shell (γ-FeO_x_@AuNP)@[C_60_(>DPAF-C_9_)_1or2_]_n_ nanoparticles (NPs). These NPs having e^−^-polarizable fullerosome structures located at the outer layer were fabricated from highly magnetic core-shell γ-FeO_x_@AuNPs. Fullerosomic polarization of **3** was found to be capable of causing a large amplification of material permittivity that is also associated with the photoswitching effect in the frequency range of 0.5‒4.0 GHz. Multilayered synthetic construction allows Förster resonance energy transfer (FRET) of photoinduced accumulative surface plasmon resonance (SPR) energy in the gold layer to the partially bilayered C_60_(>DPAF-C_9_)_1or2_-derived fullerosome membrane shell layer in a near-field of direct contact without producing radiation heat, which is commonly associated with SPR.

## 1. Introduction

Recent development of surface plasmon resonance (SPR) energy phenomena has been demonstrated and applied in many technological areas including the use of it as an alternative means to increase either light absorption or scattering in a thin film to enhance solar cells efficiency [1,2]. In general, SPR energy arises in silver or gold nanoparticles (NPs) as collective oscillation of surface electrons that is induced by the interaction with the electric field of the incident light. Light absorption is achievable when the size of NPs is controlled to be smaller than the wavelength of the incident light. It leads to polarization with the formation of polaritons. The degree of polarizability leading to the influence of absorption cross-section enhancement larger than the actual physical cross-section of the NPs can be a function of particle size and shape, refractive index, direction of electric field, and characteristics of surrounding medium [3]. For example, light absorption is proposed to be the dominate event over scattering if the particle size is smaller than ~15 nm. Gold NPs are very inefficient light emitters. Therefore, most of absorbed photoenergy is converted into irradiation heat to surrounding medium. However, if a single or arrays of gold NPs or nanometer-scaled dipole wires [4] can be fabricated, they may behave as antennas working at optical frequency [5,6,7,8] due to their strong interactions with the incident light. This is different from traditional microwave frequency antenna that is a mediator between far-field radiation and local fields (currents) in an electronic circuit.

The simplest and convenient synthetic methods for the preparation of gold nanostructures include wet chemical synthesis [9,10,11] and the sol process [12]. By synthetically creating the shape preference to increase the aspect ratio (length/width ratio) leading to anisotropic gold nanorods, their plasmon mode may become tunable upon splitting into two visible bands. This provides the possibility to tune the resonance energy band to match with dielectric materials for permittivity modulation. In addition, crystallographic facets if synthetically achievable may also exhibit different thermodynamic or photocatalytic properties from those of spherical nanoparticles. These are indicative of parameters to potentially impact materials characteristics and photoresponse ability by modification of gold nanostructures when applied in conjunction with dielectrics.

Refractive index of a given medium is a function of the product of dielectric constant or permittivity (ε) and permeability (µ). Both are relevant material parameters in response to electromagnetic waves. The former parameter is dependent on material’s degree of polarization at the wavelength of measurements. Accordingly, we designed and synthesized several electron-polarizable donor-acceptor conjugates using C_60_ cage as the acceptor moiety and highly photoresponsive, light harvesting DPAF-C_n_ chromophore as the donor antenna to construct the corresponding conjugates C_60_-(antenna)_x_ [13,14]. We also applied additional photoinduced SPR energy for the aim to cause further molecular e^‒^-polarization of such conjugates. This type of polarization arises from the induced intramolecular electron-transfer process going from the donor antenna to the e^‒^-accepting fullerene cage in an ultrafast kinetic rate in femtoseconds [15]. To achieve the near-field effect during the energy-transfer event between plasmonic Au NPs and C_60_-(antenna)_x_, we assembled a multiple layered structure of nanoparticles having an inner magnetic γ-FeO_x_@AuNP core encapsulated by an outer C_60_(>DPAF-C_9_)_x_ shell that is coupled with control of the thickness among each layer. Apparently, fabricated core-shell structural configurations are capable of inducing permittivity amplification at RF-frequency ranges.

## 2. Results and Discussion

**Figure 1:**
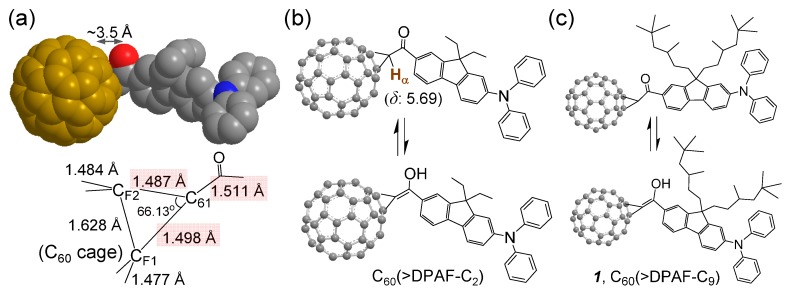
Bridging structure of a C_60_-DPAF-C_2_ conjugate with (**a**) carbon-carbon bond lengths at the C_60_ cage (C_F1_ and C_F2_) bridging unit indicated showing a shorter length of C_61_‒C(=O) than a normal single C‒C bond, as determined by X-ray single crystal structural analysis reported previously [15]; (**b**,**c**) Keto-enol tautomerism of the bridging unit structure showing a large downfielded chemical shift of *α*-proton H_α_ from the normal α-proton chemical shift of δ 2.1‒2.3. This downfield shift is also caused partly by the fullerenyl ring current.

The C_60_ cage is highly electronegative, a feature associated with its strongly electron-accepting ability. In the presence of a covalently bound electron-donating chromophore, such as 9,9-di(3,5,5-trimethylhexyl)-2-diphenylaminofluorene (DPAF-C_9_), high molecular e^‒^-polarizability characteristics can be achieved in the conjugate upon photoexcitation [16]. The phenomena arise from intramolecular charge-transfer event among e^‒^-donating DPAF-C_9_ moieties and the e^‒^-accepting C_60_, leading to the formation of a negatively charged (C_60_>)^−^**·** cage and positively charged (DPAF)^+^**·-**C_9_. In our recent studies of femtosecond transient absorption measurements, we have synthesized and presented an approach of C_60_/or C_70_-(antenna)_x_ type fullerene-chromophore dyads [14,15], triads, and pentaads [17,18] using similar C_60_/or C_70_(A)–*keto*–DPAF-C_9_(D) assembly structures. This A‒D design as that of C_60_(>DPAF-C_2_) (Figure 1) takes the advantage of both C_60_> and DPAF-C_9_ being active in linear and nonlinear photonic processes [14]. Its photoresponsive activity was verified by intramolecular energy/or electron-transfer processes and found to occur in an ultrafast rate of <130 fs [15]. In PhCN, electron-transfer process was found to dominate photophysical events giving the charge-separated state C_60_^−^**·**(>DPAF^+^**·**-C_n_)_1or2_ in a long life-time [17]. A closely linked molecular nanostructure with periconjugation between C_60_ and the carbonyl moiety of DPAF-C_n_ in a spacing distance of only ~3.5 Å [15], as shown in Figure 1a, and the concurrence of keto-enol tautomerism at the conjugation bridge in Figure 1b were reasoned for the observed ultrafast photoresponsive rate. The latter leads to the creation of partial conjugation between C_60_ and covalently linked DPAF-C_n_ separated by only one carbon atom (C_61_). This makes the photoinduced intramolecular electron-transfer very efficient and ultrafast.

**Scheme 1:**
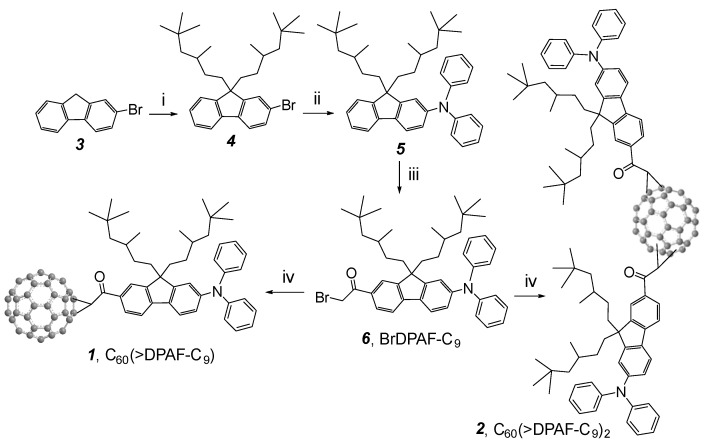
Synthesis of C_60_-(antenna)_x_ nanostructures **1** (x = 1) and **2** (x = 2) [14]. Reagents and reaction conditions: (i) 1-C_9_H_37_-OMs, *t*-BuOK in THF, 0 °C‒r.t., 4 h; (ii) diphenylamine, tris(dibenzylideneacetone)dipalladium(0) (cat.), *rac*-BINAP (cat.), *t*-BuONa, toluene, 110 °C, 8.0 h; (iii) α*-*bromoacetyl bromide, AlCl_3_, ClCH_2_CH_2_Cl, 0 °C, 4.0 h; (iv) C_60_, DBU, toluene, r.t., 5.0 h.

Synthesis of electronically polarizable push-pull conjugative structures as methano[60]ullerene dyad C_60_(>DPAF-C_9_) **1** and triads C_60_(>DPAF-C_9_)_2_ **2** was performed by using modified synthetic procedures reported recently [15], as outlined in Scheme 1 with the reagent and conditions indicated. The key precursor intermediate BrDPAF-C_9_ **6** was synthesized from 9,9-di(3,5,5-trimethylhexyl)-2-diphenylaminofluorene (**5**) with α*-*bromoacetyl bromide in the presence of Lewis acid for a period of 4.0 h. Subsequent fullerene condensation reaction was carried out by the treatment of **6** with C_60_ using DBU as a base to yield both **1** and **2** in a yield of 70 and 14%, respectively, after chromatographic purification on SiO_2_.

Spectroscopic characterization of monoadduct **1** and bisadduct **2** was made by their positive ion fast atom bombardment mass spectrum (FAB^+^‒MS) showing clearly the molecular ion mass at *m*/*z* 1345/1346 (M^+^/MH^+^) and 1971 (M^+^) for **1** and **2**, respectively. Both spectrum displayed a fragmentation peak at *m*/*z* 720/721 corresponding to the ion mass of C_60_ cage with no major detectable intermediate fragments indicating the main bond breakage occurring at the cyclopropanyl carbon conjunction to C_60_ cage.

**Figure 2:**
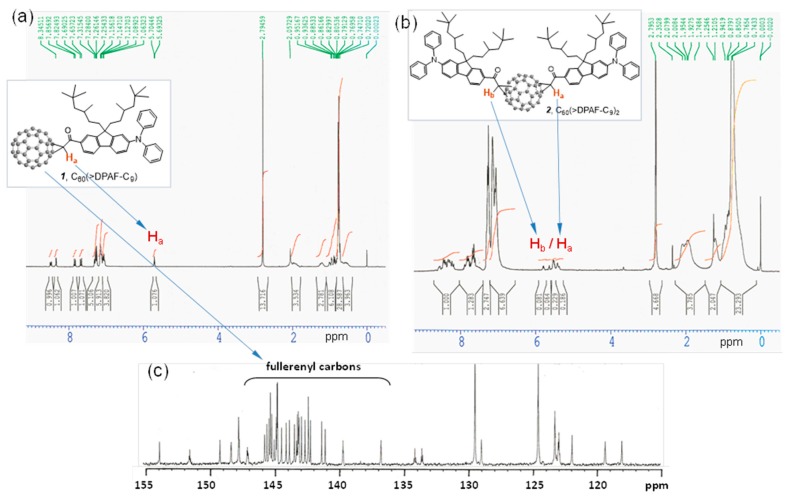
^1^H-NMR spectra of (**a**) the monoadduct C_60_(>DPAF-C_9_) **1** and (**b**) the bisadduct C_60_(>DPAF-C_9_)_2_ **2**. Chemical shift of the H_a_ proton of **1** was used to assign H_a_ and H_b_ protons of **2** that revealed only one major and one minor regioisomer existing in the chromatographically purified sample; (**c**) ^13^C-NMR spectrum of **1** indicating a *C*_2_ molecular symmetry with a total of 29 fullerenyl *sp^2^* carbons.

We assigned all major proton peaks in ^1^H-NMR spectrum of **1** and **2**. Since the compound **1** is a single component, its spectrum can be used as a reference to assist chemical shift assignments of **2**. The α-proton (H_a_ next to the carbonyl group) peak of **1** appeared as a triplet (via through space coupling with two fluorenyl protons in vicinity) at δ 5.69 (Figure 2a) with a large downfield shift of roughly 1.2 ppm from that of **6**. It was also accompanied with downfield shift of all fluorenyl phenyl protons to 7.83 (d, *J* = 8 Hz), 8.48 (d, *J* = 8 Hz), and 8.34 (s). In Figure 2b, we assigned the multiplet peaks at δ 7.7‒7.9 (2H), 8.2‒8.35 (2H), and 8.35‒8.6 (2H) to the chemical shift of three types of fluorenyl protons. There are two groups of proton peaks at δ 5.35‒5.6 and 5.6‒5.85 in a different integration intensity accounted for a total of two types of α-protons each with H_a_ and H_b_ in a slightly different chemical shift value. These revealed only two regioisomers possible for the bisadduct **2** in a molecular ratio of roughly 1:2.8 in a chromatographically non-separable fraction due to close similarity of the molecular polarity between these two regioisomers. Highly steric hindrance of DPAF-C_9_ antenna was reasoned for the isolation of a very limit number of regioisomers.

**Figure 3:**
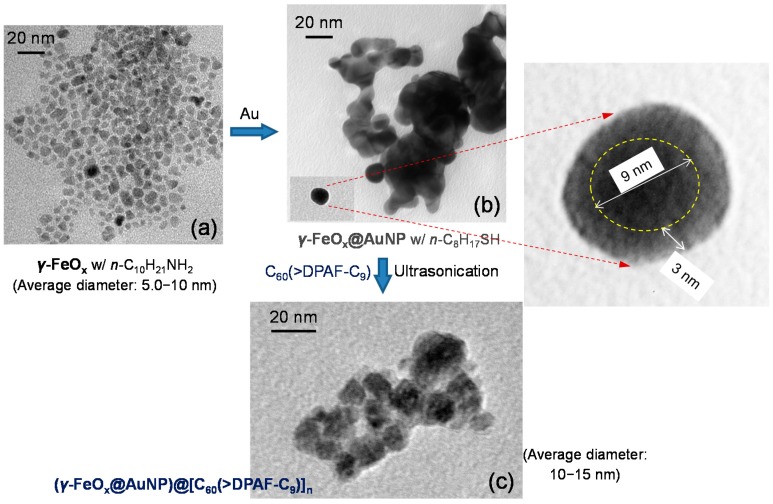
TEM micrographs of (**a**) γ-FeO_x_ (prepared by the method A1); (**b**) γ-FeO_x_@AuNP, and (**c**) C_60_(>DPAF-C_9_)-encapsulated γ-FeO_x_@AuNP nanoparticles **3**–**1** showing evolution of particle morphology changes.

Preparation of **1** or **2**-encapsulated γ-FeO_x_@AuNP trilayered core-shell nanoparticles, (γ-FeO_x_@AuNP)@[C_60_(>DPAF-C_9_)_x_]_n_ **3**–**1** (x = 1) or **3**–**2** (x = 2) was made first by the synthesis of magnetic γ-FeO_x_ nanoparticles using either the method A1 or A2 described in the experimental section. The latter method carried out at 320 °C gave monodisperse nanocrystals. The former method performed at 150 °C resulted in smaller NPs, as shown in Figure 3a. Two methods B1 and B2, as described in the experimental section, were applied for deposition of the gold layer. The latter method involves dissolution of iron oxide NPs by (CH_3_)_4_N^+^OH^‒^ in H_2_O, followed by the attachment of sodium citrate upon stirring for the reduction of hydrogen tetrachloroaurate(III) trihydrate (HAuCl_4_∙3H_2_O) along with hydroxylamine hydrochloride. Since a magnetic core NP was used as the substrate for providing a magnetic field to interact with electromagnetic waves, all coated core-shell γ-FeO_x_@AuNPs can be removed from solution easily by an external magnet and purified. Surface of the resulting gold layer was stabilized by the addition of 1-octanethiol in toluene to furnish the products of core-shell Au-nanoparticles-coated γ-FeO_x_ NPs. Sufficient binding of 1-octanethiol as a capping agent rendered moderate solubility of NPs in organic solvents ready for encapsulation by **1** and **2** under ultrasonication. The resulting relatively pure trilayered core-shell nanoparticles can be retrieved easily from the washing solution (ethanol and ether) by an external permanent magnet. The procedure provided separation of core-shell nanoparticles from the residual capping agent and an excessive amount of C_60_(>DPAF-C_9_) in solution. The binding force of **1** and **2** to the nanoparticle surface was controlled by the strong hydrophobic‒hydrophobic interaction forces of C_60_‒C_60_ cages that resulted in molecular self-assembly of C_60_(>DPAF-C_9_) to form a partial bilayer or bilayer configuration at the surface, resembling that found on the formation of bilayered *fullerosome* nanovesicles of water-soluble C_60_(>DPAF-EG_x_) reported recently [19]. Accordingly, initial interactions of **1** at the nanoparticle surface was designated to occur on two 3,5,5-trimethylhexyl moieties owing to low solubility of C_60_> in alkanes that should push fullerene cages outward at the solvent interfacial area. The sonication treatment provides sonochemical energy to invert **1** with the favor of C_60_ cage in contact with Au NPs that gives the monolayer capping of the surface by C_60_(>DPAF-C_9_) molecules [20]. They become accessible for the binding of the second layer of **1** in the solution based on strong C_60_‒C_60_ cage and C_60_‒DPAF aromatic-ring attraction forces. This is proposed to be the formation mechanism of a partial bilayered or bilayered fullerosome membrane covering γ-FeO_x_@AuNP nanoparticles as the nanostructure configuration of (γ-FeO_x_@AuNP)@[C_60_(>DPAF-C_9_)_x_]_n_ **3**.

Morphology and topography of all NPs was investigated by tunneling electron microscopic (TEM) micrographs. As a result, the average size of the parent γ-FeO_x_ nanoparticles was measured to be small as 5–10 nm in diameter (Figure 3a) in a narrow distribution. Upon deposition of a gold layer, only a slight layer contrast difference between Au and FeO_x_ was observed in Figure 3b that is still possible to differentiate a darker core iron oxide particle diameter of ~9 nm with a slightly lighter gold shell thickness of ~3.0 nm (inset of Figure 3b). Further coating of **1** at the outer shell led to encapsulation of many nanoparticles by a layer of organic substance in light soft amorphous image (Figure 3c) that was measured in a shell thickness of few nm. This thickness corresponds well to a proposed partial bilayered or bilayered packing configuration of molecules **1** on the surface of γ-FeO_x_@AuNP. It is plausible to assume the strong attraction force between the C_60_> cage and AuNP in directing the initial orientation of molecules **1** having the fullerene cage directly associated with interfacial binding on Au surface that resulted in the dialkylfluorene ring moiety facing outward to the solution media. Subsequent packing of more molecules of **1** on the layer of DPAF-C_9_ is expectable to form a layer of fullerosome membrane owing to the stronger hydrophobic–hydrophobic interaction forces of (C_60_>)–(C_60_>) than (C_60_>)–(DPAF-C_9_). By using the MM2 energy minimization technique to simulate the 3D molecular configuration with the assumption of a C_60_ cage diameter width of 1.0 nm, we estimated the molecular long-axis length of **1** in roughly 3.5 nm. This fits well with the sum of an observed bilayer packing thickness of few nm, consisting of a head-to-head orientation of **1** in the fullerosome membrane region.

**Figure 4:**
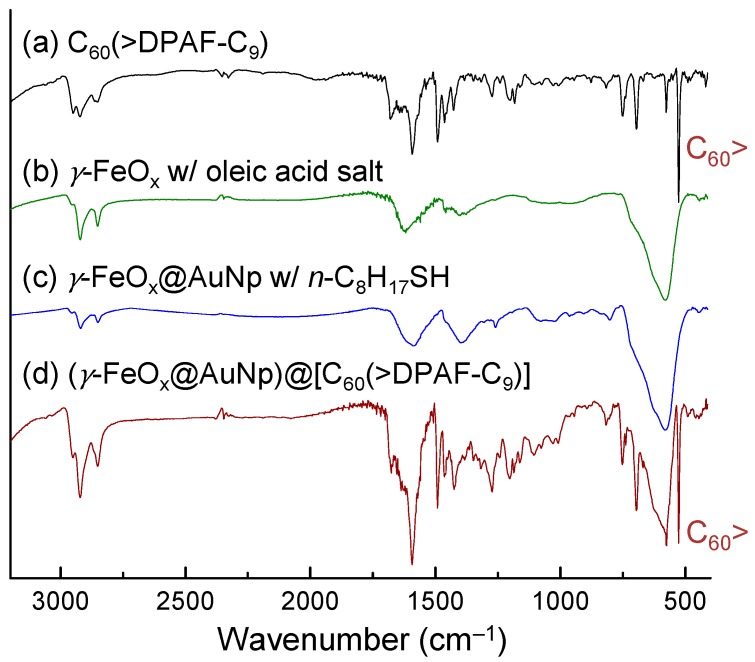
Infrared absorption spectra of four samples indicated, showing a consecutive sum of absorption bands on final product **3**–**1** in (**d**).

Successful construction of core-shell nanomaterials was verified by their infrared spectra of purified samples. Oleic acid sodium salt stabilized γ-FeO_x_ NPs (Figure 4b) displayed three medium to weak bands at 1618 (s), 1459 (w), and 1407 (m) cm^‒1^ along with the main Fe-O absorption band centered at 578 (vs) cm^‒1^. Incorporation of an Au layer on top does not change much on the main peak of spectrum in Figure 4c. Deposition of the fullerosome layer of **1** on the outer layer obviously incurred major absorption band changes in Figure 4d to show carbonyl (C=O) bands at 1741 (m), 1675 (m), and 1637 (m) cm^‒1^ that revealed various bonding interactions of at least two moieties, one from oleic acid and the other carbonyl group in solid state of **1**. Olefin (C=C) absorption band of **1** centered at 1591 cm^‒1^ can be detected very clearly. The most indicative strong sharp band at 526 cm^‒1^ of Figure 4d can be assigned to the half-cage absorption of C_60_> that is consistent with that of **1** in Figure 4a.

**Figure 5:**
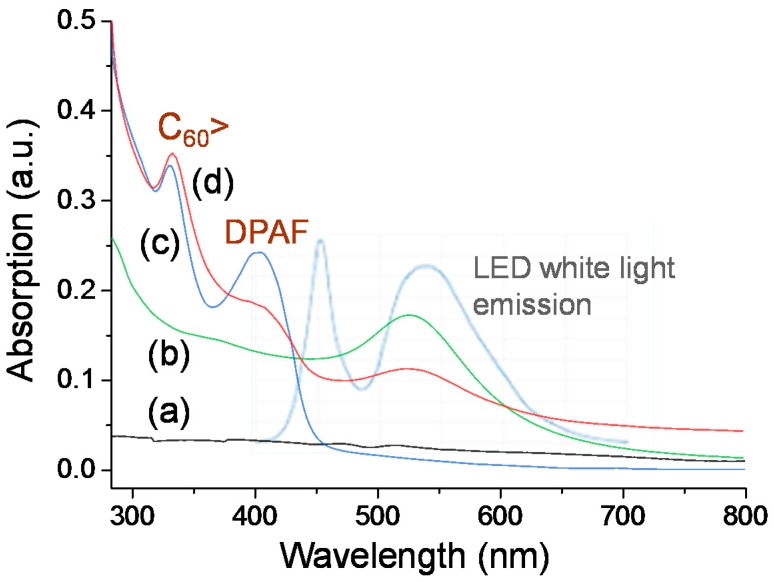
UV-vis absorption of (**a**) γ-FeO_x_ NPs stabilized by *n*-C_10_H_21_NH_2_; (**b**) γ-FeO_x_@AuNP stabilized by *n*-C_8_H_17_SH; (**c**) C_60_(>DPAF-C_9_) **1** in CHCl_3_; and (**d**) **3**–**1** in CHCl_3_‒THF (1:5) with their absorption correlation to emission bands (**light blue**) of white LED light used.

Optical absorption of the Au nanoparticle subshell layer is crucial for this study to allow us executing and introducing photoinduced surface plasmon resonance energy generation at UV to visible light wavelengths (300‒550 nm), followed by subsequent near-field SPR energy-transfer from the inner layer to the outer C_60_(>DPAF-C_9_)_x_-derived fullerosome layer. This is in addition to the ability of **1** or **2** to perform photoinduced intramolecular charge-separation themselves to the corresponding transient C_60_^−^**·**(>DPAF^+^**·-**C_9_) state for **1** and C_60_^−^·(>DPAF^+^**·-**C_9_)(>DPAF-C_9_) or C_60_^−2^(>DPAF^+^**·-**C_9_)_2_ states for **2** at the same wavelength range covering roughly 250‒380 nm and 350‒430 nm corresponding to absorptions of C_60_> and DPAF-C_9_ moieties, respectively. These absorption bands remain unchanged during the fabrication procedure of **3**–**1**, as shown in comparison between Figure 5c,d). The broad absorption band (Figure 5b) of the Au layer covers the most of the spectrum wavelength range at 280–630 nm with the extinction coefficient higher than that of γ-FeO_x_ (Figure 5a).

**Figure 6:**
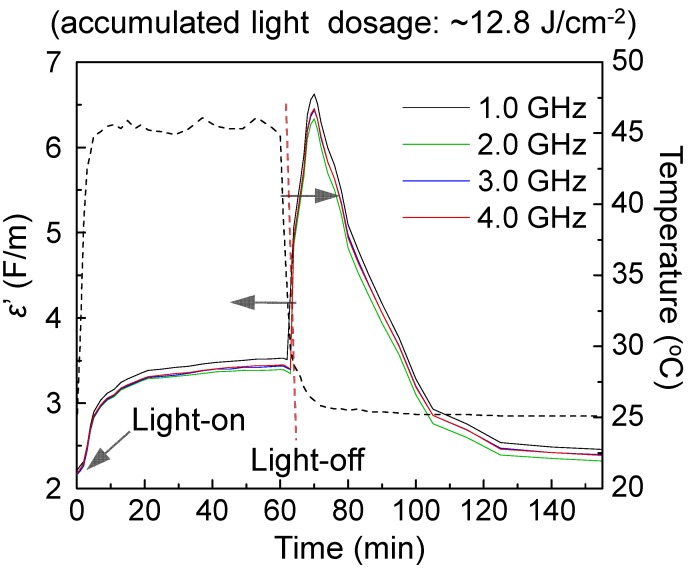
Time-dependent relative dielectric constant (ε_r_′) profiles of trilayered sample **3**–**1** at the frequency range of 1.0‒4.0 GHz under illumination using a LED white light, showing large photoswitchable amplification of *ε*_r_′ at the light-off state.

Frequency-dependent dielectric and permittivity measurements of **3**–**1** and **3**–**2** were carried out using an open-ended coaxial probe and a network analyzer in the range of 0.5‒4.5 GHz to study properties including the relative dielectric constant (ε_r_′, the real part of complex permittivity) and the relative dielectric loss factor (ε_r_′′, the imaginary part of complex permittivity). It was conducted inside a custom-built chamber to create a circumferentially uniform illumination environment. Inner chamber temperature was maintained and controlled using several fans. Illumination periods were chosen to be 10, 30, and 60 min. The objective of these measurements was to investigate the dielectric amplification of nanoparticles in various core-shell layered configurations in response to external photoinduced generation of SPR effects within the gold-shell layer. We selected a LED white light with the output power of 2.0 W and a UVB-enhanced visible lamp with the output power density of 20 mW/cm^2^ for the study. With the use of a white light, the optical absorption should occur mainly on the gold layer since the absorption peak maximum (λ_max_) of C_60_> cage and DPAF-C_9_ moieties are centered at 326 and 410 nm, respectively. Figure 6 shows relative dielectric constant (ε_r_′) curves of **3**–**1**, as an example, with the data taken at the frequency of 1.0‒4.0 GHz during and after 60 min illumination. The inner chamber temperature was monitored constantly during the irradiation period at the sample tube wall. In all experiments, white light illumination led to a slight rise in temperature to 39‒52 °C in the first 10 min. Increase of chamber temperatures from 25 °C resulted in corresponding small increases of the ε_r_′ value of samples in a different extent, as shown in Figure 6. The value remains relatively constant during further irradiation from 10 to 60 min. In separate similar experiments using three reference samples of **1** alone, γ-FeO_x_ NPs, and core-shell γ-FeO_x_@[C_60_(>DPAF-C_9_)]_n_ NPs without the Au layer, no detectable large change of ε_r_′ values was observed during or after the irradiation period. Therefore, observed large ε_r_′ changes with the core-shell sample **3**–**1** can be correlated to the ε_r_′ value increase of C_60_(>DPAF-C_9_)-derived fullerosome membrane, as a result of photoinduced plasmon-energy generation in the gold layer.

The sharp increase of permittivity can be accounted by a ratio of ε_max_′/ε_o_′ (ε_o_′ = 2.25 at 1.0 GHz and time zero) in a roughly 2.89-fold or 1.91-fold (ε_max_′/ε_r_′, based on ε_r_′ = 3.4 at 60 min) at the epak maximum reached at roughly at the time of 70 min (10 min after the start of light-off state). This is considered to be a novel switch-on permittivity phenomenon that we have observed to date. We hypothesized that the release of SPR energy from the intermediate gold layer sandwiched between the layers of fullerosome membrane and core γ-FeO_x_ Np caused activation of the molecular polarization of C_60_(>DPAF-C_9_)_x_ molecules to its transient charge-separate (CS) state. This CS state consists of a positively charged electron-donor moiety (DPAF)^+^**∙-**C_9_ and a negatively charged electron-acceptor moiety (C_60_>)^‒^** -**. This is in a large contrast to no clear change of ε_r_′ values of core-shell nanoparticles without incorporation of an Au-layer at the light switch-off stage. Subsequently, the amplified ε_r_′ underwent a relaxation process back to the initial values within the next 20 min, revealing good recyclability and photoswitchability during light-on and light-off cycles.

## 3. Experimental Section

### 3.1. Materials

Reagents and solvents: aluminum chloride, 1,2-dichloroethane, α*-*bromoacetyl bromide, sodium sulfate, 1,8-diazabicyclo[5.4.0]undec-7-ene (DBU), FeCl_3_·6H_2_O, FeCl_2_·4H_2_O, sodium acetate, *n*-octylamine, 1,2-hexadecanediol, 1-oleylamine, 1,2-propanediol, sodium oleate, 1-oleic acid, 1-octadecene, Au(ac)_3_, hydrogen tetrachloroaurate(III) trihydrate (HAuCl_4_∙3H_2_O), phenyl ether, tetramethylammonium hydroxide, 1-octanethiol, and hydroxylamine hydrochloride were purchased from Aldrich Chemicals (St. Louis, MO, USA) and used without further purification. All other chemicals were purchased from Acros Chemicals Ltd. (New Brunswick, NJ, USA). The anhydrous grade solvent of THF was refluxed over sodium and benzophenone overnight and distilled under reduced pressure (10^−1^ mmHg).

### 3.2. Spectroscopic Measurements

^1^H-NMR and ^13^C-NMR spectra were recorded on either an Avance Spectrospin–200 or AC-300 spectrometer (Bruker, Billerica, MA, USA). UV-vis spectra were recorded on a U-3410 UV spectrometer (Hitachi, Chiyoda, Tokyo, Japan). Infrared spectra were recorded as KBr pellets on a 750 series FT-IR spectrometer (Thermo Scientific Nicolet, Waltham, MA, USA). Photoluminescence (PL) spectra were measured using PTI Fluorescence Master Systems (Photon Technology International, Edison, NJ, USA) connected with a photomultiplier (914 Photomultiplier Detection System) with a xenon short arc lamp as the excitation source. Mass spectroscopic measurements were performed by the use of positive ion matrix-assisted laser desorption ionization (MALDI–TOF) technique on a M@LDI-LR mass spectrometer (Micromass, Cary, NC, USA). The sample blended or dissolved in the matrix material was irradiated by nitrogen UV laser at 337 nm with 10 Hz pulses under high vacuum. Mass ion peaks were identified for the spectrum using the MassLynx v4.0 software. In a typical experiment, the samples of **5**, **6**, and **7**, were dissolved in CHCl_3_ in a concentration of 1.0 mg/mL. The matrix of 3,5-dimethoxy-4-hydroxycinnamic acid (sinapic acid) was dissolved in THF in a concentration of 10 mg/mL. The solution of matrix (1.0 mL) was taken and mixed with the sample solution (0.1 mL) prior to the deposition on a stainless-steel MALDI target probe. It was subsequently dried at ambient temperature.

### 3.3. Synthetic Procedures

Synthesis of 7-α-Bromoacetyl-9,9-di(3,5,5-trimethylhexyl)-2-diphenylaminofluorene BrDPAF-C_9_ (**6**). A similar method as that of a recently reported procedure was used [14].

Synthesis of 7-(1,2-Dihydro-1,2-methanofullerene[60]-61-carbonyl)-9,9-di(3,5,5-trimethylhexyl)-2-diphenylaminofluorene, Monoadduct C_60_(>DPAF-C_9_) (**1**) and Bisadduct, C_60_[methanocarbonyl-7-(9,9-di(3,5,5-trimethylhexyl)-2-diphenylaminofluorene)]_2_, C_60_(>DPAF-C_9_)_2_ (**2**). A similar method as that of a recently reported procedure was used [14]. Spectroscopic data of **2**: FAB^+^‒MS calcd for ^12^C_150_^1^H_110_^14^N_2_^16^O_2_ *m*/*z* 1970.8; found, *m*/*z* 1971; MALDI−MS (TOF) found, *m*/*z* 1974, 1973, 1972, 1971 (M^+^), 1347, 1346, 870, 861, 710, 705, 672, 666, 630, 629, 612, and 583; UV-vis (CHCl_3_, 2.0 × 10^‒5^ M) λ_max_ (ε) 255 (1.9 × 10^5^), 310 (1.1 × 10^5^), and 406 nm (8.5 × 10^4^ L/mol-cm); FT-IR (KBr) *ν*_max_ 3429 (br), 3163, 2951 (s), 2922, 2863, 1680, 1595 (vs), 1492 (vs), 1465, 1420, 1400, 1316(w), 1277, 1200, 1156 (w), 1093, 1030 (w), 816 (w), 753, 670 (s), and 527 (s) cm^‒1^; ^1^H-NMR (500 MHz, CDCl_3_, ppm) δ 8.5−8.1 (m, 4H), 7.9−7.4 (m, 4H), 7.24 (m, 12H), 7.12 (m, 8H), 7.05 (m, 4H), 5.8−5.3 (m, 2H), 2.1−1.8 (m, 8H), 1.35−1.15 (m, 8H), and 1.1−0.5 (m, 60H). ^13^C-NMR (500 MHz, CDCl_3_, ppm) δ 189.8, 154.1, 151.8, 151.7, 149.4, 148.1, 148.0, 147.7, 147.5, 146.9, 146.8, 146.6, 146.2, 146.0, 145.9, 145.7, 145.5, 145.0, 145.0, 144.9, 144.8, 144.7, 144.5, 144.3, 144.0, 143.9, 143.7, 143.6, 143.3, 143.3, 143.1, 143.0, 143.0, 142.9, 142.6, 142.5, 142.3, 142.3, 142.2, 142.1, 140.9, 140.5, 140.0, 139.8, 139.7, 139.5, 134.5, 134.0, 129.8, 129.5, 129.3, 128.65, 124.9, 123.6, 122.3, 119.6, 118.5, 73.4, 73.2, 55.6, 51.3, 51.2, 44.4, 44.4, 42.9, 38.2, 38.2, 38.1, 33.6, 33.5, 33.5, 31.4, 30.4, 29.9, 27.7, 23.1, and 22.9.

#### 3.3.1. Synthesis of γ-FeO_x_ (1.0 < x < 1.5) Nanoparticles

*Method A1*. In a typical reaction, a mixture of FeCl_3_·6H_2_O (2.70 g, 0.010 mol), FeCl_2_·4H_2_O (1.98 g, 0.010 mol), sodium acetate (4.92 g, 0.060 mol, as a hydrolyzing agent), H_2_O (4.0 mL), and *n*-octylamine (7.3 mL, as a capping agent) in 1,2-propanediol (66 mL) were prepared and stirred under reflux at 150 °C for a period of 5.0 h. It was followed by precipitation upon the addition of 2-propanol to afford the product in a nearly quantitative yield. Spectroscopic data: FT-IR (KBr) ν_max_ 2970 (w), 2924 (s), 2852 (m), 1743 (w), 1630 (s), 1458 (m), 1383 (m), 1134 (m), 1041 (m), 922 (w), 879 (w), 839 (w), 627 (vs), and 584 (vs) cm^‒1^.

*Method A2*. Iron oleate was firstly prepared by heating iron chloride, FeCl_3_·6H_2_O (5.4 g, 20 mmol) and FeCl_2_·4H_2_O (2.0 g, 10 mmol), and sodium oleate (36.5 g, 120 mmol) at 70 °C in a mixture solvent, composed of ethanol (80 mL), distilled water (60 mL), and hexane (140 mL), for a period of 4.0 h. At the end of reaction, the upper organic layer containing the iron oleate complex was washed three times with distilled water (30 mL each), dried over sodium sulfate, and solvent evaporated off to yield iron oleate complex in a waxy solid form. To this resulting organometallic complex, 1-oleic acid (4.3 g, 15 mmol) and 1-octadecene (200 g, act as the solvent) were added at room temperature and refluxed at 320 °C for 1.5 h. Upon the temperature reaching 320 °C, a severe exothermic reaction occurred with the initial transparent solution turning to turbid and brownish black. The product mixture was cooled to room temperature, followed by precipitation with ethanol (500 mL). Resulting nanoparticles were collected using a permanent magnet, washed twice with both 2-propanol (100 mL) and ethanol (100 mL). They were re-dispersed in nonpolar solvent, such as hexane and toluene, via ultrasonication. In general, dark solid particles of γ-FeO_x_ (Fe_3_O_4_) obtained by this method were found to be highly magnetic in response to the external magnet and soluble in many nonpolar organic solvents. Distribution of the particle size is fairly homogenous and was measured via TEM as ~20 nm in diameter on average. Spectroscopic data: FT-IR (KBr) *ν*_max_ 2960 (w), 2921 (s), 2852 (m), 1618 (s), 1459 (w), 1407 (m), and 578 (vs) cm^‒1^.

#### 3.3.2. Synthesis of Au-Nanoparticles Coated γ-FeO_x_ Nanoparticles, γ-FeO_x_@AuNP

*Method B1*. Our first trial was involving the direct reduction and deposition of gold acetate, Au(ac)_3_, on the surface of iron oxide NPs (10‒20 nm, prepared by either methods A1 or A2) in the presence of 1,2-hexadecanediol and 1-oleylamine. Briefly, to the suspension of γ-FeO_x_ NPs (0.1 g) in phenyl ether (20 mL) was added Au(ac)_3_ (0.45 g, 1.2 mmol), 1,2-hexadecanediol (3.0 g, 25 mmol), and oleylamine (4 mL) under N_2_ atmosphere. The mixture was heated to 180‒190 °C with vigorously stirred for 2.0 h while maintain the temperature constant. After cooling to room temperature, the NPs were collected by a permanent magnet, washed twice with both 2-propanol (100 mL) and ethanol (100 mL). Subsequently, the NPs were collected with a permanent magnet to yield a dark brown solid powder.

*Method B2*. To the γ-FeO_x_ nanoparticles (prepared by the method A1 stated above, 100 mg) was added D.I. water (100 mL) and tetramethylammonium hydroxide (1.0 M, 10 mL, 10 mmol). The mixture was ultrasonicated for 1.0 h to dissolve particles and yield a transparent brown solution. The solution was filtered through 0.4-μm PTFE syringe filter to remove insoluble large particles. The solution was then diluted to 2.0 L by D.I water, added by sodium citrate (0.5 M, 100 mL, 50 mmol), and stirred for 30 min at ambient temperature. While kept stirring, the solution of hydroxylamine hydrochloride (1.0 M, 2.0 mL, 2.0 mmol) and hydrogen tetrachloroaurate(III) trihydrate (HAuCl_4_∙3H_2_O, 0.025 M, 2.0 mL, 0.05 mmol) were added portionwise in 1/10 quantity each by an interval period of 15 min. After finishing addition, a magnet was used to separate and remove the particles containing γ-FeO_x_ from the solution. They were washed with D.I water (×2) and ethanol (×2) and, finally, washed with methanol and decanted the supernatant. The suspension solution was then added acetone (10 mL), methanol (10 mL), and 1-octanethiol (1.0 mL) and ultrasonicated for 1.0 h to replace hydrophilic stabilizer (tertiary amine or sodium citrate) on the surface by hydrophobic thiol hydrocarbons. The resulting solids were filtered and washed by water and ethanol to remove remaining residual hydrophilic stabilizers and excessive 1-octanethiol to yield core-shell Au-NPs coated γ-FeO_x_ nanoparticles. Spectroscopic data: FT-IR (KBr) ν_max_ 2958 (m), 2928 (s), 2852 (m), 1724 (w), 1626 (s), 1496 (m), 1463 (s), 1380 (m), 1330 (w), 1263 (w), 1168 (m), 1130 (s), 1078 (s), 877 (w), 804 (m), 730 (s), 696 (m), 609 (m), 584 (s), and 557(m) cm^‒1^.

#### 3.3.3. Preparation of C_60_(>DPAF-C_9_)-Encapsulated γ-FeO_x_@Au Trilayered Core-Shell Nanoparticles, (*γ*-FeO_x_@AuNP)@[C_60_(>DPAF-C_9_)]_n_

We carried out an encapsulation procedure by dissolving γ-FeO_x_@AuNP nanoparticles (100 mg) in toluene with the aid of a small quantity of 1-octanethiol, followed by the addition of C_60_(>DPAF-C_9_) (100 mg) and subsequent sonication for a period of 30 min. After filtration of the solution through celite, an excessive amount of C_60_(>DPAF-C_9_) (est. in <5 mg) remaining in the solution was removed. All encapsulated magnetic nanoparticles filtered off at the top celite surface as brown solids were recovered by toluene extraction and physically removed from the solution by an internal permanent magnet. Solids were washed repeatedly by ethanol and ether, followed by drying in vacuo.

Alternatively, a modified method was used to prevent the possible material loss during celite filtration. In this procedure, both γ-FeO_x_@AuNP nanoparticles (100 mg) and C_60_(>DPAF-C_9_) **1** or C_60_(>DPAF-C_9_)_2_ **2** in a predefined weight ratio amount were dissolved in toluene (30 mL) with stirring for 30 min and then ultrasonicated for an additional more than 30 min until a clear solution being obtained to homogenize nanoparticles dispersion and increase their interactions with **1** or **2**. The solution was concentrated via rotary evaporation to less than 3.0 mL in volume to increase the molecular contact of **1** or **2** to γ-FeO_x_@AuNPs. It was then diluted by toluene again to a volume of 30 mL with stirring and subsequent sonication for 10 min to dissolve excessive **1** or **2**. All encapsulated magnetic nanoparticles were physically removed from the container solution with the assistance of an internal permanent magnet. The nanoparticles were washed repeatedly by ethanol and ether, followed by drying in vacuo to afford dark brown solids of (γ-FeO_x_@AuNP)@[C_60_(>DPAF-C_9_)_x_]_n_ (x = 1 or 2) **3**. Spectroscopic data of **3**–**1** prepared based on the nanoparticle methods A1 and B2: FT-IR (KBr) *ν*_max_ 2952 (m), 2920 (s), 2848 (m), 1741 (m), 1675 (m), 1637 (m), 1618 (m), 1591 (s), 1490 (m), 1458 (s), 1429 (m), 1382 (m), 1271 (m), 1203 (s), 1168 (m), 1110 (m), 1076 (m), 1029 (m),.875 (w), 811(w), 748 (m), 694 (m), 617 (m), 582 (s), and 526 (s) cm^‒1^.

### 3.4. Tunneling Electron Microscopic (TEM) Measurements

Both formvar and carbon-copper film grids in a 200-mesh size were used for the topography investigation of molecularly assembled structures, derived from **1** and **2**, by TEM images. Samples were prepared by direct coating the grid with a sample solution of 1.0 × 10^−6^ M diluted from the master nanoparticle solution (5.0 × 10^−4^ M in H_2_O). Different concentrations (10^−4^–10^−6^ M) may be used and evaluated to ensure full separation of particles from each other without stacking prior to the collection of microimages. It was followed by the freeze-dry technique via placing the sample-coated grid on a metal dish that was in thermal equilibrium with liquid nitrogen. This apparatus was then placed inside a vacuum chamber for overnight to remove all solvents completely prior to the measurement that will retain the nanoparticle shape on the grid. To prevent particle aggregations during the solvent removal procedure, a low concentration of 10^−5^–10^−6^ M in either H_2_O or benzene is essential.

### 3.5. Dielectric and Permittivity Measurements

Dielectric property measurements were carried out by an Agilent Network Analyzer (Agilent Technologies, Inc., Santa Clara, CA, USA) equipped with an open-ended Agilent 85070E dielectric probe kit (200 MHz to 50 GHz). Calibration was conducted by using open-ended, close-ended, and attenuated calibrators prior to each measurement to remove the cable-related instability and system drift errors. In the experiment, a complex scattering parameter, defined as S_11_, was measured and converted to relative complex electric dielectric constant values using Agilent 85071E Materials Measurement Software. This complex form is compdose of a real and an imaginary part. The former represents the value of dielectric constant and the latter is defined as the loss factor.

Two light sources were used in the measurement including a LED white light with the output power of 2.0 W and a UVB-enhanced visible lamp (Uvitron 400 W with the output power density of 20 mW/cm^2^). Poly(dimethylsiloxane) (PDMS, 1.0 g) semi-solid was applied as a polymer matrix host that is capable of forming a paste-like sample with **2**. A mixture of PDMS and either C_60_(>DPAF-C_9_), γ-FeO_x_ NPs, γ-FeO_x_@AuNPs, or (γ-FeO_x_@AuNP)@[C_60_(>DPAF-C_9_)]_n_ (100 mg) was prepared by dissolving both components in ethyl acetate (20 mL) in the testing tube under sonication until a clear solution was obtained. Ethyl acetate was then completely evaporated under vacuum to yield brown paste-like semi-solid materials.

A custom-built chamber was applied for permittivity measurements to be conducted under a circumferentially uniform illumination environment. It was achieved by the installation of a reflective half-circular aluminum plate at the back-wall side surrounding the testing tube which is located at the center of the chamber. A light was allowed to pass through a small window at the front side of the chamber to irradiate the sample tube. Reflected light beams were design to refocus from the back-side aluminum mirror plate back to the tube. Four fans were installed at the top and side-walls of the chamber to control and prevent temperature building-up inside the chamber. Illumination periods were chosen to be 10, 30, and 60 min.

## 4. Conclusions

We design and synthesized several analogous nanomaterials by sequential construction of three-layered core-shell (*γ*-FeO_x_@AuNP)@[C_60_(>DPAF-C_9_)_1or2_]_n_ **3** nanoparticles. These NPs consisting of an electron-polarizable, bilayered fullerosome membrane located at the outer layer of a NP were fabricated by the attachment of photoresponsive C_60_(>DPAF-C_9_)_1or2_ molecules on highly magnetic core-shell γ-FeO_x_@AuNPs. Highly e^‒^-polarizable fullerosome of **3** was found to be capable of producing a charge-separated state of **1** as C_60_^‒^**∙**(>DPAF^+^**∙-**C_9_) that causes a large amplification of material permittivity. The observed phenomena are also associated with the photoswitching effect in the frequency range of 0.5‒4.0 GHz. Apparently, multilayered synthetic construction of a core-shell NPs makes inter-layer interactions much more effective in a near field of direct contact. This allows photoinduced accumulative plasmon resonance energy in the gold layer to effect Förster resonance energy transfer (FRET) to the partially bilayered fullerosome membrane shell layer without producing much radiation heat. The occurrence was followed by the relaxation of both ε_r_′ and ε_r_′′ back to their original values that revealed an excellent recyclability. We suggest that fullerosomic array of C_60_ cages in the solid state may be useful in providing a pathway and mechanism for long-lived charge-delocalized (C_60_>)^n−^ (n ≥ 1) charges among nanocages in the nanomembrane, leading to large permittivity increases.
